# Transphyseal Resection of Post-Traumatic Linear Physeal Bars of the Distal Tibia Using a Large-Diameter Trephine: A Report of Two Cases

**DOI:** 10.3390/pediatric18050136

**Published:** 2026-10-12

**Authors:** Vladimir Kenis, Anastasia Shubina

**Affiliations:** 1 H. Turner National Medical Research Center for Children’s Orthopedics and Trauma Surgery, Parkovaya 64–68, Pushkin, 196603 Saint-Petersburg, Russia; 2 North-Western State Medical University Named After I.I. Mechnikov, 191015 Saint-Petersburg, Russia

**Keywords:** physeal bar, distal tibia, growth plate, bar resection, temporary hemiepiphysiodesis, guided growth, children

## Abstract

Background: Physeal fractures of the distal tibia may result in persistent growth arrest and physeal bar formation. An asymmetrical bar, combined with continued skeletal growth, leads to progressive ankle deformity. Physeal bar resection is a standard procedure for local growth plate damage. However, linear bars that traverse the entire sagittal plane from the anterior to the posterior cortex are technically challenging to curette. Methods: Two clinical cases of pediatric patients with posttraumatic linear distal tibia bars are presented. Both patients underwent resection using a large-diameter trephine and bone wax. Temporary hemiepiphysiodesis with an 8-plate was also performed, although this proved technically unsuccessful in one case. Results: Both patients underwent transphyseal resection of a linear physeal bar of the distal tibia using a 12 mm trephine, followed by temporary hemiepiphysiodesis. One patient experienced proximal migration of the epiphyseal screw and plate displacement, which was managed conservatively. At final follow-up, joint axis was restored (MDTA improved from 72° to 92° in Patient 1 and from 75° to 91° in Patient 2 at 4.5 and 4 years after surgery, respectively). Conclusions: Our findings demonstrate that transphyseal bar resection of post-traumatic linear physeal bars of the distal tibia using a large-diameter trephine can serve as the technical option for correction of varus ankle deformity in growing children. In both patients, correction of varus ankle deformity was observed after the combined procedure—trephine resection with bone-wax interposition and temporary lateral hemiepiphysiodesis. Because both patients underwent concomitant guided growth, the relative contribution of bar resection and hemiepiphysiodesis cannot be determined from these two cases.

## 1. Introduction

Distal tibia fractures are among the most common skeletal injuries in children [1,2]. Traumatic physeal injury may result in localized premature physeal closure and formation of a physeal bar, with clinically significant growth disturbance occurring in approximately 5% of patients within two years of injury [3,4]. In children with substantial remaining growth, this leads to progressive axial malalignment. A medially located bar typically produces varus deformity due to relatively unopposed lateral physeal growth. The reported incidence of growth disturbance after distal tibial physeal fracture ranges from 15% to 16% [5,6].

Physeal bar resection is the standard treatment for focal lesions involving up to 50% of the physis, most often performed after distal femoral fractures and less commonly after proximal tibial fractures [7]. Resection of a distal tibial bar is technically and prognostically challenging: the fracture line runs predominantly in the sagittal plane across the entire diameter of the distal epiphysis, and the resulting bar extends from the anterior to the posterior cortex. Curettage of such a long, narrow bar through a conventional metaphyseal window is difficult, and completeness of resection is hard to confirm.

This article presents two cases of post-traumatic linear physeal bars of the distal tibia treated with transphyseal resection using a large-diameter trephine, bone-wax interposition, and temporary lateral hemiepiphysiodesis. The procedure was performed under general anesthesia with fluoroscopic guidance. The bar was localized with a guidewire, and a small skin incision corresponding to the trephine diameter was made. Resection was performed using a 12 mm trephine directed in the anteroposterior plane. The guidewire was advanced under lateral fluoroscopy through the center of the bar and intentionally crossed the posterior cortex by 1–2 mm to serve as a trajectory reference. Its advancement was controlled by lateral fluoroscopy. The trephine was then advanced over the guidewire. The trephine tip was advanced until it reached and perforated the posterior cortical margin. Its advancement was stopped immediately after cortical perforation under continuous lateral fluoroscopic control. No additional protective instrument was placed posterior to the tibia. Soft-tissue protection consisted of limiting penetration under fluoroscopic depth control. The walls of the residual cavity were coated with bone wax. The procedure was supplemented by temporary hemiepiphysiodesis with an 8-plate placed on the lateral aspect of the distal growth plate.

## 2. Presentation of the Cases

Patient 1, an otherwise healthy girl, at the age of 8.5 years, she sustained a Salter-Harris type II fracture of the distal tibia while skiing. As the displacement was considered minor, open reduction was not performed. She was immobilized in a plaster cast for 6 weeks, followed by physical therapy with weight-bearing. One year after the injury, the patient presented to our clinic due to the progression of a varus deformity of the ankle. Over the next 6 months, the deformity increased. Radiography and CT scan revealed a linear bar in the sagittal plane in the medial part of the growth plate of the distal tibia.

The size of physeal bar involvement was quantified on preoperative CT. Length was measured on sagittal CT reconstruction as the maximum anteroposterior extent of the bar at the level of the physis, from the anterior to the posterior physeal margin. It does not include extra-physeal extension. Because the physis has an irregular shape and the variation in its height exceeds the slice thickness, we selected a plane on 3D rendering that was close to the axial plane and in which the bar area was maximal and performed measurements on that slice. Total physeal area was delineated on this slice tracing the entire cross-section of the bone and cartilage along the outer margin; bar area was defined as the dense area within that cross-section. The ratio was expressed as a percentage. Additionally, given predominantly linear shape of the bar, the width of the physeal bar was measured in the widest point. The outer cutting diameter of the trephine was 12 mm. Its wall thickness was 1.5 mm, so the internal diameter was 9 mm. The resected specimen diameter was therefore approximately 9 mm. Because the widest portion of the bar measured 8 mm, we anticipated complete resection of the bar within the trephine.

Passive and active range of motion was symmetrical in varus position (joint plane) but limited (geometrically) when assessed in relation to the limb axis.

**Figure 1:**
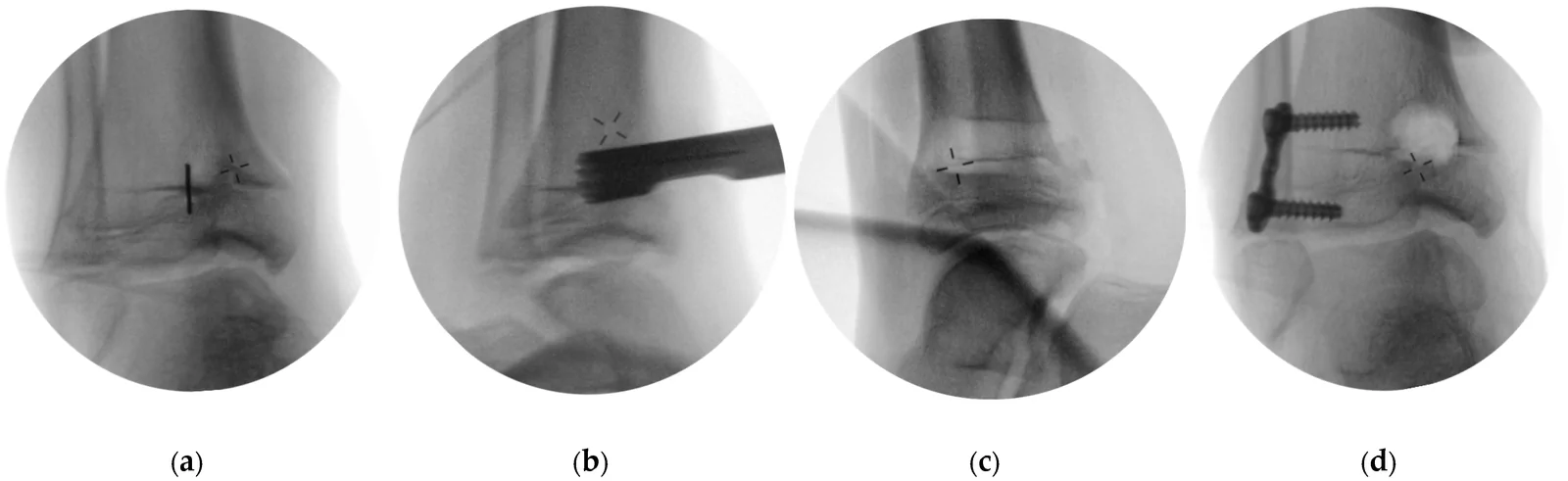
Intraoperative stages of transphyseal bar resection in patient 1: (**a**) placement of a guidewire in the anteroposterior plane; (**b**) advancement of the 12 mm trephine over the guidewire under fluoroscopic control; (**c**) the resected bone cylinder containing the physeal bar; (**d**) immediate postoperative anteroposterior radiograph showing the resection cavity and 8-plate.

Given the progressive varus deformity and open physes, operative intervention was indicated. Due to the technical difficulties of curettage of linear bars in this area, resection was performed with a large-diameter trephine (12 mm outer diameter of the instrument was chosen given the widest cross-section of the bar 8 mm) (Figure 1a–c).

A 12 mm trephine was introduced through an anterior metaphyseal window under fluoroscopic guidance. The guidewire was passed through the center of the bar and to exit just beyond the posterior cortex to serve as a reference for the trephine trajectory. The posterior cortical margin was perforated and soft tissues were protected by limiting the trephine advancement under lateral fluoroscopic control.

The targeted bar appeared to be removed on fluoroscopy and by inspection of the resected specimen and the visible portion of the channel. Given depth and small diameter of the channel complicating direct visualization, we additionally inspected the resected bony cylinder confirming presence of the cartilaginous strip alongside both borders. Bone wax was applied to the inner walls of the channel by direct application.

The 8-plate was implanted on the lateral part of the distal tibia, immediately anterior to the fibula (Figure 1d). Postoperatively, the limb was immobilized in a brace for 3 weeks, with partial weight-bearing at 1 month and full weight-bearing at 8 weeks.

**Figure 2:**
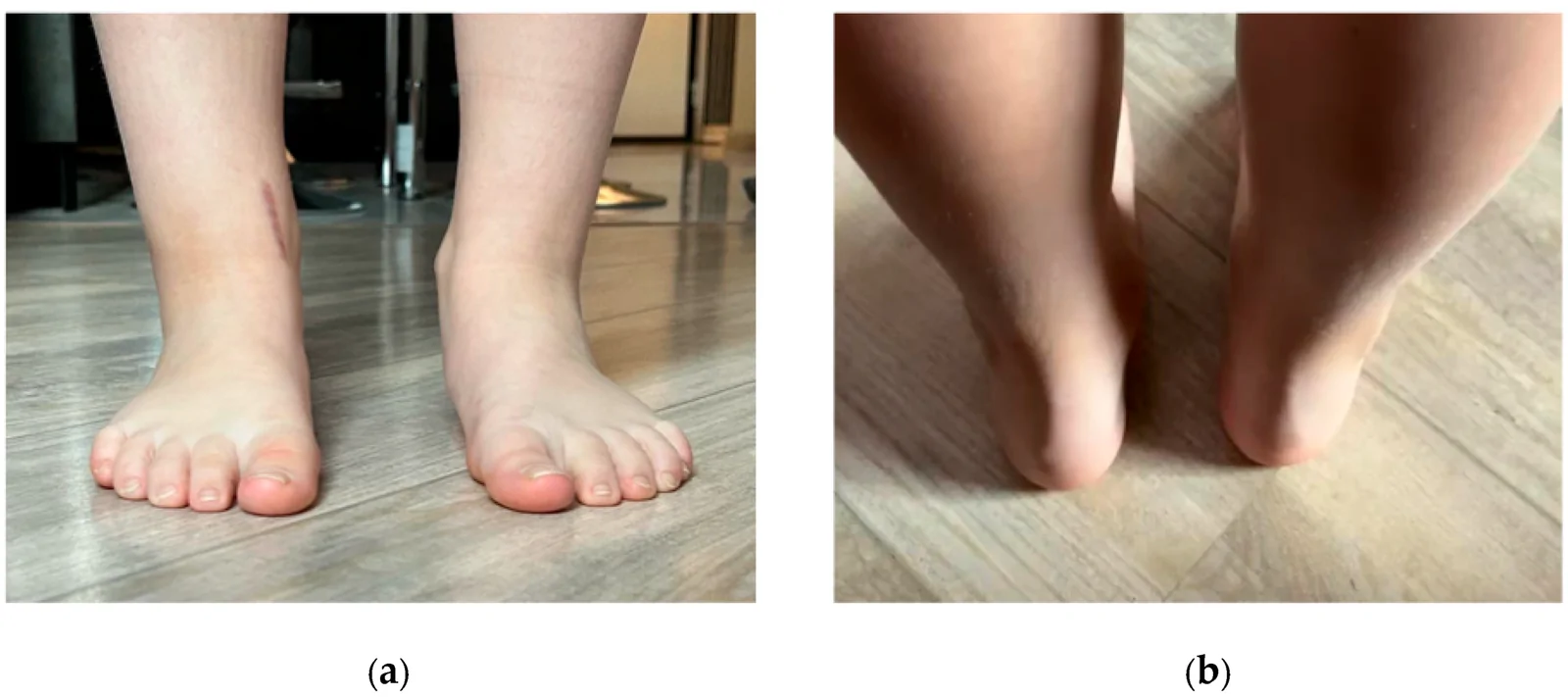
Clinical photographs of Patient 1 at final follow-up: (**a**) frontal view and (**b**) posterior view showing hindfoot alignment and minimal anterior scar.

**Figure 3:**
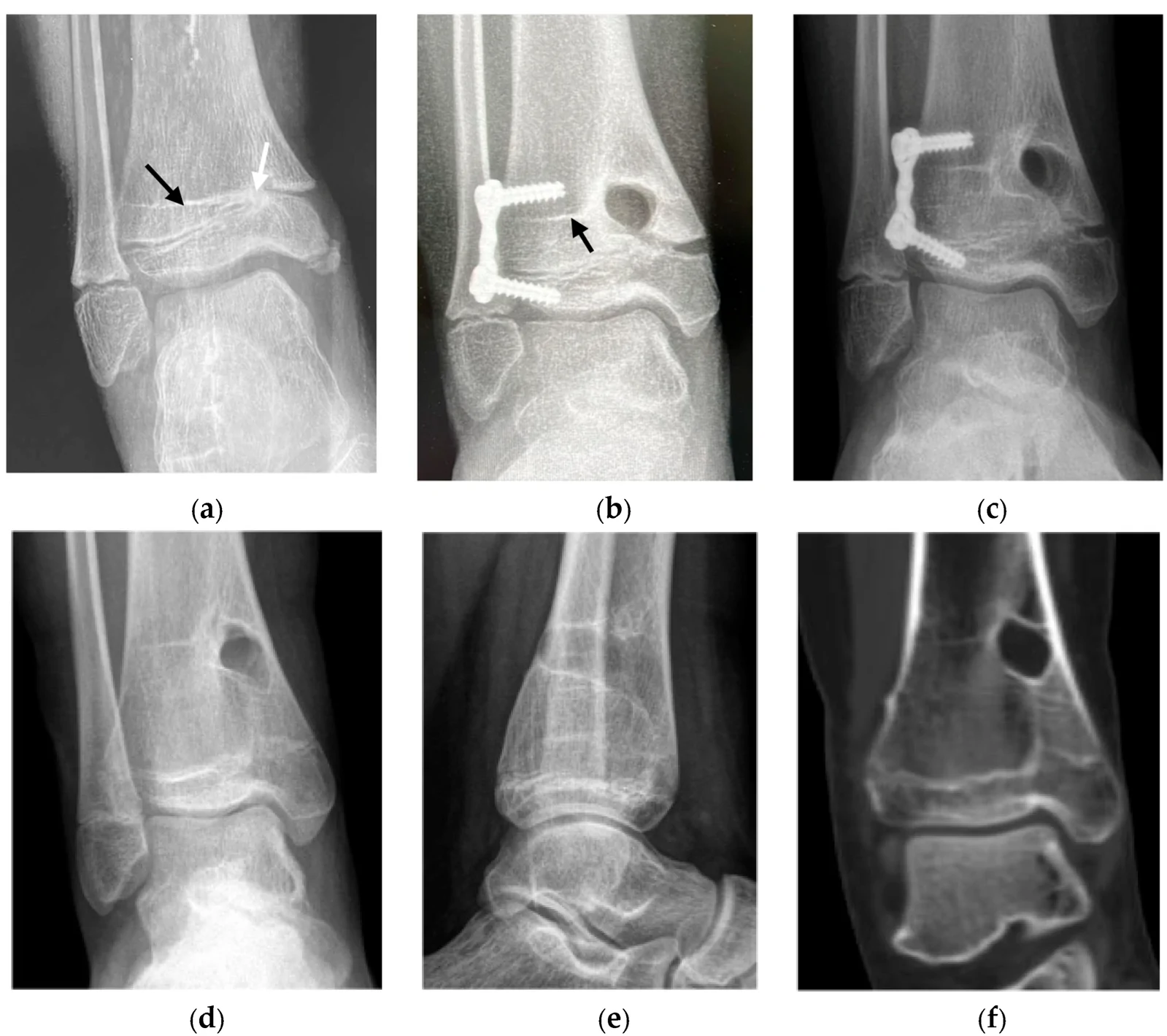
Radiographic and computed tomographic evolution of deformity correction in patient 1: preoperative imaging (**a**) shows a medial longitudinal physeal bar (white arrow) with converging Harris line (black arrow) and a varus deformity of the ankle; at 1 year (**b**), early correction, screw divergence, and parallelization of Harris line (black arrow) are observed; at 2 years (**c**), the ankle joint line is horizontal, and the resection cavity has migrated away from the physis; at 4 years (**d**), the resection cavity lies at the diaphyseal level, with spontaneous correction of the fibular axis; the lateral radiograph (**e**) and coronal CT (**f**) at 4 years confirm the persistent cavity and a horizontal articular surface.

Correction of the deformity was already noted on the control radiographs 1 year after surgery. Complete correction of the varus deformity was achieved after 30 months. The 8-plate was removed after correction was achieved. The patient remains under follow-up at 2 years after plate removal (Figure 2 demonstrates clinical photographs at final follow-up 4.5 years postoperatively), with maintenance of the radiographically confirmed correction (Figure 3).

Patient 2, a girl with cerebral palsy (level I according to Gross Motor Function Classification System). She had previously undergone conservative management for a dynamic equinovarus deformity, including treatment with botulinum toxin. She sustained an inversion foot injury on the paretic left side, which was not promptly or accurately assessed due to her underlying condition and pre-existing neurogenic varus position. Radiographs were taken one week later, and Salter-Harris type IV distal tibial fracture was diagnosed. Closed reduction and plaster immobilization were performed. Varus deformity was noted immediately after cast removal, and it progressed over the next year. A post-traumatic linear physeal bar was identified in the medial aspect of the distal tibial growth plate.

**Figure 4:**
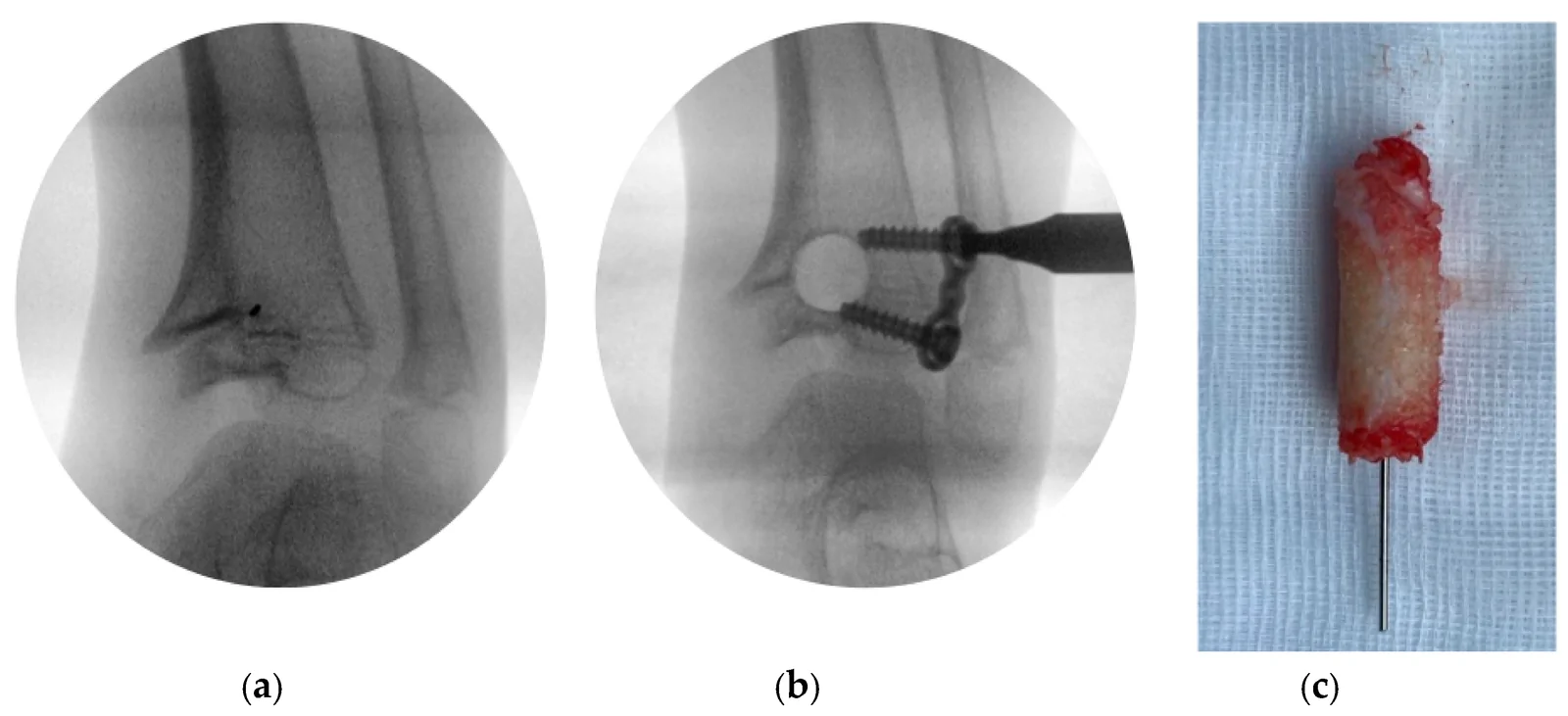
Surgical stages for patient 2: (**a**) guidewire placement along the sagittal trajectory of the bar; (**b**) the 8-plate secured to the lateral tibia; (**c**) the resected specimen, confirming visible physeal margins.

**Figure 5:**
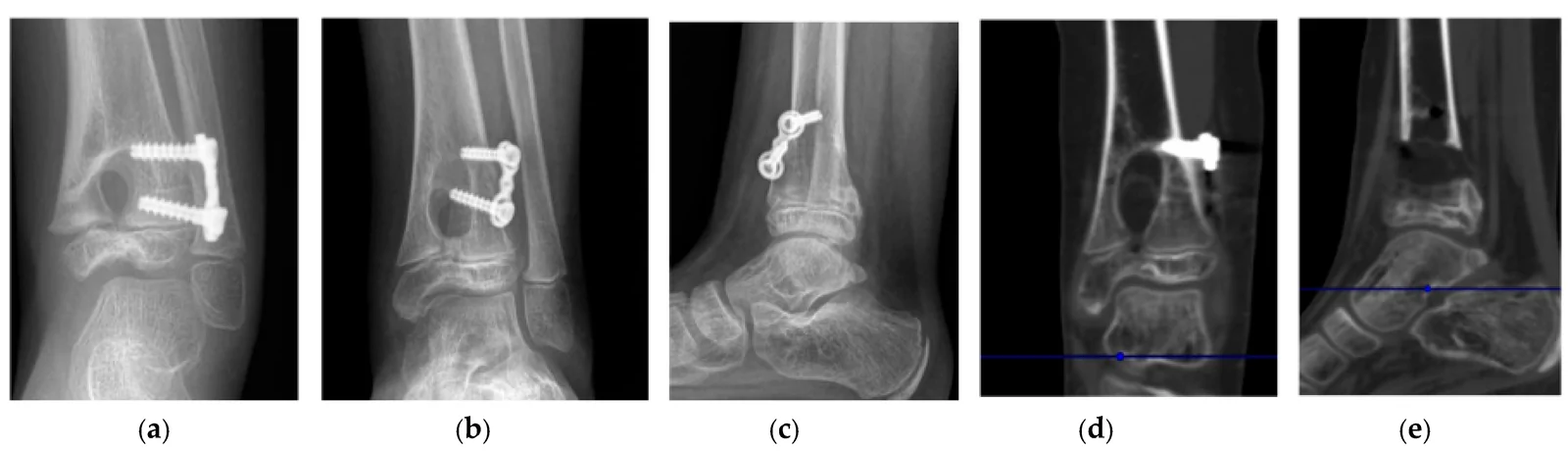
Radiographic and CT dynamics in patient 2: (**a**) at 1 year, the resection cavity has migrated from the physis, the epiphyseal screw shows proximal migration; (**b**) at 2 years, correction of the ankle joint line and fibular alignment is evident; (**c**) lateral radiograph at 2 years demonstrates the transphyseal canal and proximal plate migration; (**d**) coronal CT at 2 years shows a horizontal joint line; (**e**) sagittal CT at 2 years confirms the anteroposterior orientation of the residual canal.

**Figure 6:**
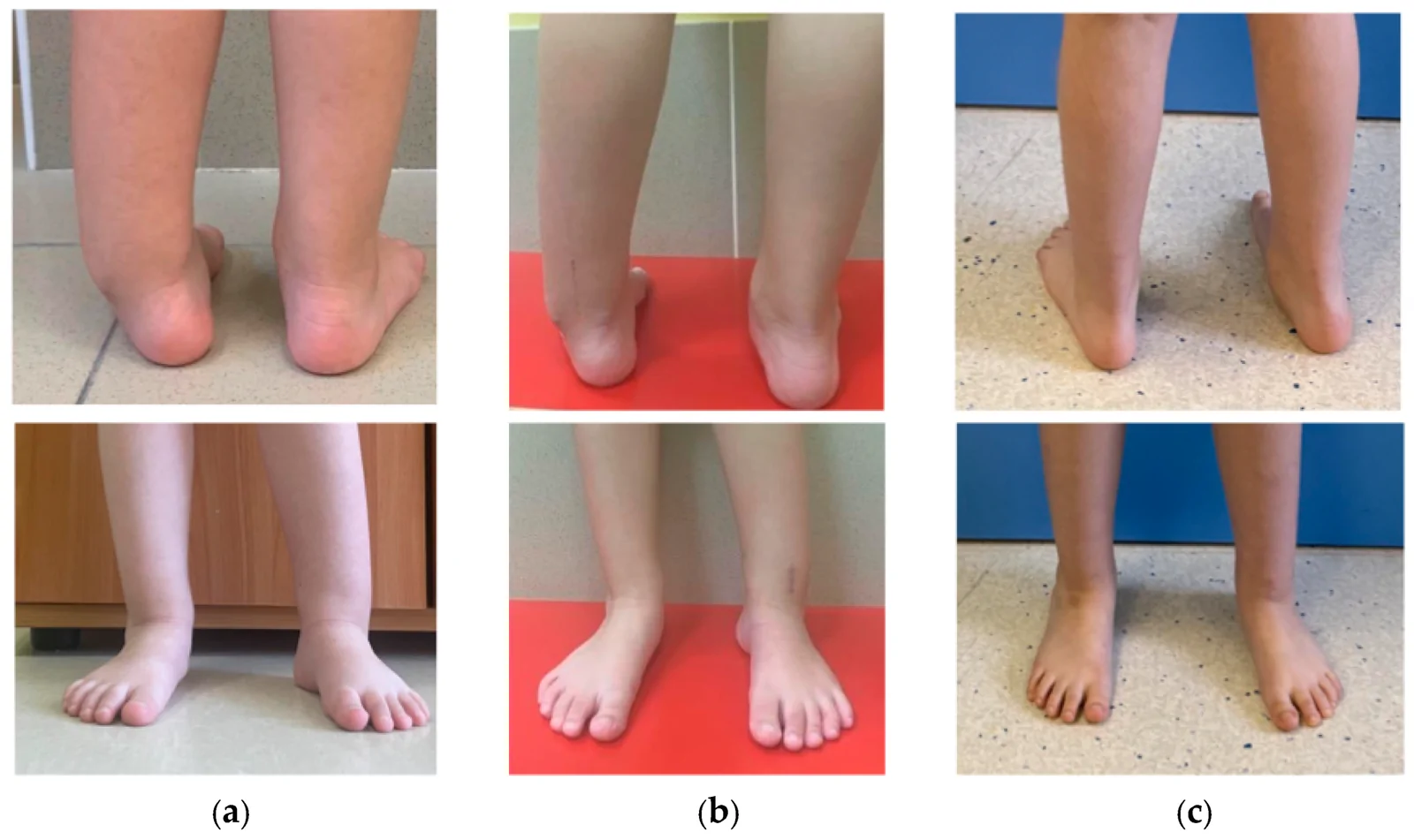
Clinical appearance of patient 2: preoperative views (**a**), views at 1 year postoperatively (**b**); views at 4 years postoperatively (**c**).

Given the patient’s high functional potential (independent walking), as well as the “double trouble” of spastic varus component and post-traumatic varus deformity, a decision was made to perform surgical treatment: bar resection with a trephine and temporary hemiepiphysiodesis with 8-plate (Figure 4a,b). The bar was resected as completely as could be judged from the specimen and visible channel (Figure 4c), and the walls were covered with bone wax. The same approach and postoperative protocol as in Patient 1 were used in this patient. Radiographs obtained six months postoperatively revealed malpositioning of the epiphyseal screw and proximal migration of the plate. However, since initial correction was observed, plate replacement was not performed. Radiographic and CT dynamics in 1 year revealed that the resection cavity has migrated from the physis (Figure 5a–c). CT at 2 years after surgery confirmed the residual canal (Figure 5d,e). After 24 months, the 8-plate was removed; no re-intervention was required (Figure 6). The patient has been followed for two years after plate removal (4 years after surgery). The treatment results are maintained.

The medial distal tibial angle (MDTA) was measured on weight-bearing anteroposterior ankle radiographs as the angle between the longitudinal axis of the tibia and the articular surface of the distal tibial plafond. Clinical hindfoot alignment was assessed clinically as the angle between the longitudinal axis of the tibia and the axis of the hindfoot on standing. All radiographic measurements were performed by an independent radiologist and retrieved from the medical records; clinical measurements were made by the first author (V.K.). The preoperative varus deformity (MDTA) was 72° in Patient 1 and 75° in Patient 2. Correction to 93° and 91° was achieved at 30 and 24 months after surgery, respectively. Limb-length discrepancy was 15 and 7 mm preoperatively and 10 and 5 mm at final follow-up, respectively. Estimated remaining growth was calculated retrospectively using the Paley multiplier method for girls, based on chronological age and actual tibial length at the time of surgery (URL https://www.limblength.org/about-us/physician-education/multiplier-mobile-app; accessed on 7 October 2026) (Table 1). This calculation provides an estimate of total remaining tibial growth as 3.8 cm in Patient 1 and 6.7 cm in Patient 2 (Table 1). The decision to perform temporary lateral hemiepiphysiodesis was based on chronological age, the presence of open physes, the degree and progression of varus deformity, and clinical judgment, rather than on a formal remaining-growth calculation.

Ankle passive dorsiflexion and plantarflexion were assessed clinically preoperatively and at final follow-up. Limb-length discrepancy was measured preoperatively on full-length standing anteroposterior radiographs as the difference in tibial length between the involved and contralateral sides. At subsequent follow-up visits, it was assessed clinically to avoid additional radiation exposure.

**Figure 7:**
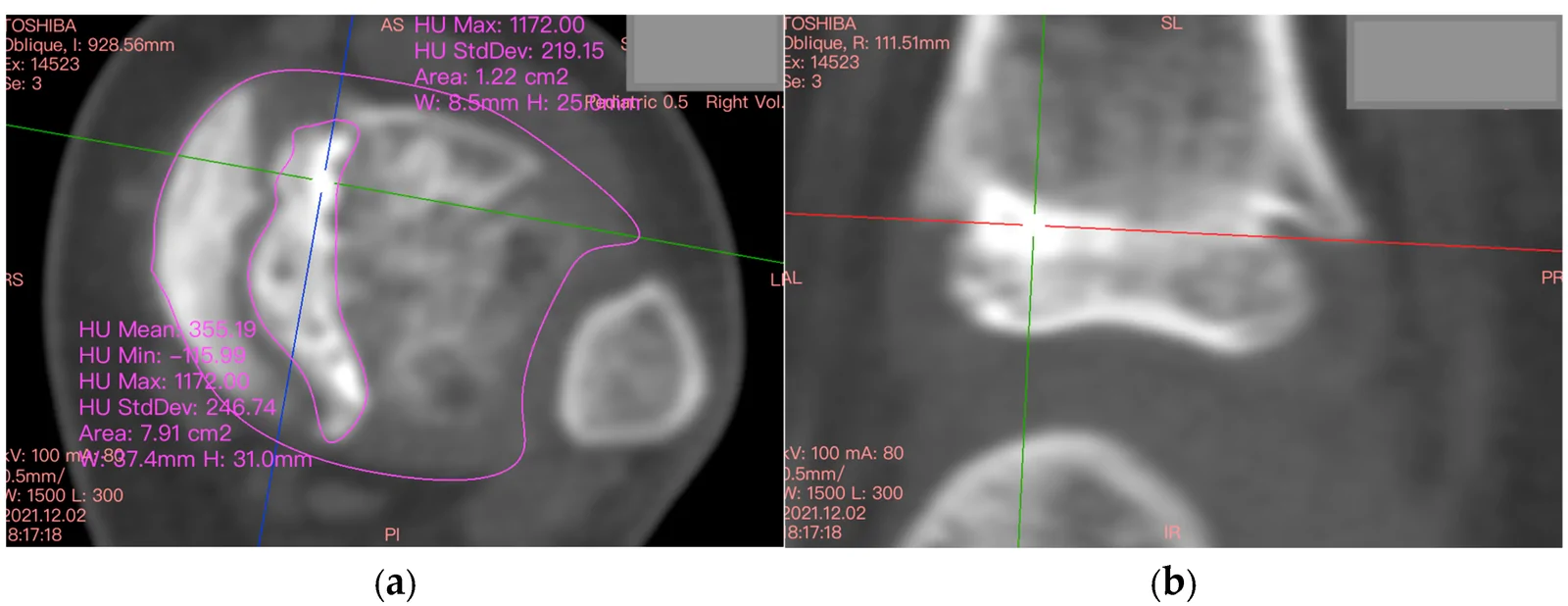
Visualization and measurement of the bar on the multiplanar CT reconstruction of patient 2 in axial (**a**) and sagittal view (**b**) demonstrating the principle of measurement of the physeal bar area as the ratio of the bar area to the physis area.

The physeal bar involved 17% of the physeal area in Patient 1 and 15% in patient 2, CT (axial view) was used for measuring and surgical planning in both patients (Figure 7). Although these values should be interpreted with caution given planar distortions and actual the 3-dimensional nature of both the bar and physis.

## 3. Discussion

This report describes two pediatric cases of post-traumatic linear physeal bars of the distal tibia treated with transphyseal resection using a large-diameter trephine, bone-wax interposition, and temporary lateral hemiepiphysiodesis. The sequelae of transphyseal fractures—growth disturbance and secondary deformity—are well described [8,9,10]. The incidence of bar formation varies with treatment method, age, and injury severity, and the management of growth-plate injuries remains controversial [11,12,13]. Growth disturbances related to epiphysiodesis predominantly manifest as varus deformities, and surgical correction is generally recommended for ankle deformities ≥ 10 degrees [6].

Bar resection is a standard procedure for focal physeal bar, but evidence for its effectiveness specifically in the distal tibia is limited [14]. Some authors have cautioned against combining guided growth with bar resection because of the risk of excessive tethering of the normal growth plate [15]. The anatomical characteristics of a linear bar make curettage difficult and may require a relatively extensive intervention [16]. In contrast to the often centrally located distal femoral bars, which are accessible through a metaphyseal window, distal tibial fractures typically produce a sagittal-plane fracture line running through the entire cross-section of the epiphysis and metaphysis from anterior to posterior. Even a small residual displacement can therefore lead to a linear physeal bar spanning the full anteroposterior dimension of the bone. Although such a bar may occupy a small cross-sectional area, its substantial length exerts a pronounced effect on limb alignment during subsequent growth. Its eccentric medial position further increases varus deformity. Historically, technical limitations and concerns about poor outcomes led some authors to question bar resection at this location [17]. However, modern imaging, fluoroscopy, and instrumentation have improved outcomes, and resection is now recommended in selected cases [18].

We employed transphyseal resection with a large-diameter trephine in both patients. Correction of deformity was observed in both cases, although the contribution of isolated bar resection cannot be determined. Screw divergence in Patient 1 is compatible with guided-growth activity, but it does not quantify the contribution of the 8-plate to the observed correction.

In Patient 2, radiographs obtained six months postoperatively revealed malposition of the epiphyseal screw and proximal migration of the plate. This was considered a technical complication rather than a planned isolated resection. A malpositioned plate cannot be assumed to exert zero biomechanical tethering or corrective force across the physis during growth. Our observation can be assumed as a hypothesis: isolated resection may be sufficient in selected patients with substantial remaining growth, but this cannot be proven from the present cases. The younger age and greater remaining growth potential of Patient 2 may have contributed to the favorable outcome, but the concomitant presence of the plate prevents any definitive attribution.

In discussions with the parents, the primary focus was the progressive varus deformity rather than limb-length discrepancy, since the latter cannot be reliably predicted. The remaining-growth calculations presented here were performed retrospectively.

Bone wax was selected as an interposition material because it is readily available, provides hemostasis, and acts as a mechanical barrier; it is widely used in physeal bar resection surgery [13,19,20]. The choice of interposition material remains a matter of discussion, with authors reporting materials ranging from autologous fat to bone cement. We acknowledge that this debate extends beyond the scope of a two-case report. The persistent osseous canal observed on late imaging may be partly related to the trephine defect itself rather than to bone wax alone.

In Patient 2, proximal migration of the epiphyseal screw and plate displacement occurred. The option of revision (removal or repositioning of the plate) was discussed with the parents. However, at the next follow-up examination, correction of the deformity was observed. It was therefore decided to continue observation with a plan to reposition the plate if the correction proved insufficient. The malpositioned plate may have continued to exert some tethering or corrective effect. The rationale for avoiding immediate revision was to avoid further physeal damage, infection, and additional anesthesia while correction was progressing.

Screw fixation within the narrow distal tibial epiphysis in a young child presents technical challenges. Strategies to improve fixation in thin epiphyseal bone include meticulous intraoperative fluoroscopic confirmation of screw placement and careful selection of plate position. This complication highlights the difficulties of guided growth in this anatomical region.

Almost all published techniques for physeal bar resection describe either marginal resection of a peripherally located bar or curettage through a metaphyseal cortical window. The latter approach presents several technical problems: the angle of instrument access, difficulty manipulating instruments within the bone when the bar is long, and difficulty assessing completeness of resection. Various visualization adjuncts, from dental mirrors to video endoscopes, have been employed [21], and osteotomies have been used to improve exposure [22]. Computer-assisted navigation and patient-specific guides have also been introduced to improve resection accuracy [23]. Our technique combines the advantages of a marginal approach with transphyseal instrument placement: the trephine is inserted from the bone edge and advanced to the opposite cortex, including the central portion of the bar. The skin incision need only accommodate the instrument; in our cases it did not exceed 3 cm. The technique is intended to achieve complete resection of the macroscopically visible bar; however, the intraoperative observations cannot definitively establish that no residual bar tissue remained. In both patients, resection of the macroscopically visible bar was suggested by intraoperative fluoroscopy, inspection of the resected bone cylinder, and direct visualization of the accessible portion of the transphyseal channel. However, these assessments cannot definitively exclude residual bar tissue. The trephine creates a narrow, deep channel, and the posterior part of the channel as well as the physeal walls beyond the trephine diameter cannot be directly visualized. Examination of the resected specimen confirmed that the targeted cartilaginous strip had been included, but it does not prove that all bar tissue within the physis was removed. This limitation is relevant when interpreting the observed correction: residual bar tissue could have contributed to persistent tethering, although the progressive correction suggests that a sufficient amount of the bar was removed to allow growth. Future studies using intraoperative endoscopy, navigation, or higher-resolution imaging may improve confirmation of resection completeness.

We believe the closest technique to ours is that reported by authors who used a large-diameter reamer (18 mm) to remove a central distal femoral bar in a three-year-old child [24]. Because of the large defect, they filled it with bone cement; however, they did not aim to remove the bar across the entire transverse diameter of the metaphyseal cartilage.

Preoperative CT examinations were performed at referring institutions and were available for review. The original rationale for selecting CT rather than MRI is unknown to us. Meanwhile, MRI can also be used for these reasons in the further studies to avoid excessive radiation exposure.

The decision to add temporary lateral hemiepiphysiodesis was based on the degree of varus deformity (MDTA 72° in Patient 1 and 75° in Patient 2), the potential remaining growth, and the expected rate of correction from guided growth. In Patient 2, the pre-existing neurogenic varus component also influenced the decision.

There is a potential risk of iatrogenic damage to the adjacent healthy physis when advancing a large-diameter trephine across the growth plate. In our cases, the guidewire was centered within the bar under multiplanar fluoroscopy, the trephine was advanced slowly without levering, and the trajectory was confirmed before full resection. In narrow bars, a smaller trephine or navigation may be preferable. Fluoroscopy and inspection of the excised cylinder were used to confirm that the macroscopically visible bar had been resected. However, because the deep portion of the resection channel could not be directly visualized, residual bar tissue cannot be definitively excluded. Although the trephine inevitably removes a rim of healthy physeal tissue adjacent to the bar, we did not quantify the total affected physeal area. This lack of quantitative assessment of the healthy physeal tissue removed is also a limitation of the present study.

A 12 mm trephine was used for bars measuring 7–8 mm in width, which implies that a strip of healthy physis was removed along both sides of the bar over its full sagittal length. The total proportion of the physis removed by the trephine, including the bar and the adjacent healthy physeal tissue, was therefore greater than the bar area alone. The trephine diameter should be matched as closely as possible to the widest cross-section of the bar to minimize unnecessary removal of healthy physis.

A limitation of this report is the small sample size and the lack of a control group, which precludes definitive conclusions. The malpositioned plate in Patient 2 is a further limitation: it prevents us from drawing conclusions about the efficacy of isolated bar resection. We report this technical complication because it reflects real-world challenges of hardware placement in this anatomical region and provides material for discussion regarding combined versus isolated techniques. Passive dorsiflexion and plantarflexion were not limited in Patient 1 and were functionally limited in Patient 2 because of the underlying neurological condition. The presence of GMFCS Level I cerebral palsy in Patient 2 also confounds direct interpretation which also lead to the difference between radiographic deviation calculated from the MDTA and clinical hindfoot alignment secondary to the pre-existing neurogenic varus before trauma. We did not perform instrumental biomechanical analysis and histopathological analysis of the resected tissues in these two patients; 3D gait analysis and pedobarography would be valuable in future studies.

## 4. Conclusions

A linear post-traumatic physeal bar of the distal tibia can be resected using a large-diameter trephine as part of a combined treatment strategy. The malpositioned plate in one patient confounds the assessment of isolated bar resection, and the present two cases cannot establish that isolated resection provides equivalent correction. These findings should be regarded as preliminary and hypothesis-generating. Further studies with larger cohorts and longer follow-up are required to refine the indications and prognostic factors for this combined approach.

## Figures and Tables

**Table 1:** Summary of patient characteristics, physeal bar parameters, treatment, and radiographic outcomes in two pediatric cases.

Parameter	Patient 1	Patient 2
Demographics and timeline		
Age at trauma, years	8.5	6.5
Age at surgery, years	10	8
Age at plate removal, years	12.5	10
Age at final follow-up, years	14.5	12
Follow-up after surgery, years	4.5	4
Follow-up after plate removal, years	2	2
Radiographic alignment (MDTA)		
MDTA at surgery, °	72	75
MDTA at plate removal, °	93	91
MDTA at final follow-up, °	92	91
Varus deformity at surgery (deviation from 90°), °	18	15
Clinical hindfoot alignment and ankle range of motion		
At surgery	15° varus	20° varus
At plate removal	5° valgus	Neutral
At final follow-up	5° valgus	Neutral
Ankle dorsiflexion	20°	15°
Ankle plantarflexion	45°	45°
Physeal bar characteristics		
Imaging modality	CT	CT
Length, mm	41	32
Width (max), mm	8	7
Physeal area involved, %	17%	15%
Location	Medial, sagittal	Medial, sagittal
Limb length (measurements and discrepancy)		
Involved tibia length, mm	255	215
Estimated total remaining tibial growth, cm	3.8	6.7
Limb length discrepancy at surgery, mm	15	7
Limb length discrepancy at plate removal, mm	10	5
Limb length discrepancy at final follow-up, mm	10	5

## Data Availability

De-identified data supporting the findings of this study are available from the corresponding author upon reasonable request.

## References

[1] Kraus R., Kaiser M. (2008). Growth disturbances of the distal tibia after physeal separation--what do we know, what do we believe we know? A review of current literature. Eur. J. Pediatr. Surg..

[2] Sapogovskiy A.V., Kenis V.M., Agranovich O.E., Trofimova S.I., Abramov I.A., Petrova E.V., Kasev A.N. (2025). Inversion injury of ankle joint and foot in children: Association with sports participation and comorbidities (analysis based on medical information system data). Pediatr. Traumatol. Orthop. Reconstr. Surg..

[3] Yuan B.J., Stans A.A., Larson D.R., Peterson H.A. (2019). Excision of Physeal Bars of the Distal Femur, Proximal and Distal Tibia Followed to Maturity. J. Pediatr. Orthop..

[4] Yamamura M.K., Carry P.M., Gibly R.F., Holmes K., Ogilvie B., Phillips A., Georgopoulos G., Miller N.H., Payne K.A. (2023). Epidemiology of Physeal Fractures and Clinically Significant Growth Disturbances Affecting the Distal Tibia, Proximal Tibia, and Distal Femur: A Retrospective Cohort Study. J. Am. Acad. Orthop. Surg..

[5] Chen H., Chen Z., Chen P., Zheng Z., Lin J. (2022). Incidence of growth disturbance after distal tibia physeal fracture in children. J. Orthop. Surg. Res..

[6] Blondin E., Stourbe O., Plancq M.C., Deroussen F., Gouron R., Klein C. (2022). Outcomes of pediatric distal tibial physeal fractures. Orthop. Traumatol. Surg. Res..

[7] Khoshhal K.I., Kiefer G.N. (2005). Physeal bridge resection. J. Am. Acad. Orthop. Surg..

[8] Schurz M., Binder H., Platzer P., Schulz M., Hajdu S., Vécsei V. (2010). Physeal injuries of the distal tibia: Long-term results in 376 patients. Int. Orthop..

[9] Pearce R., Markes A., Katyal T., Siu J., Swarup I. (2024). Outcomes After Salter-Harris II Distal Tibia Fractures in Children. Children.

[10] Leary J.T., Handling M., Talerico M., Yong L., Bowe J.A. (2009). Physeal fractures of the distal tibia: Predictive factors of premature physeal closure and growth arrest. J. Pediatr. Orthop..

[11] Peterson H.A. (1994). Physeal fractures: Part 3. Classification. J. Pediatr. Orthop..

[12] Peterson H.A. (2011). Physeal bar excision: A case report with a forty-two-year follow-up. J. Bone Jt. Surg..

[13] Alhassan M.S., Park K.B., Kim H.W., Park H., Park K.H. (2024). The efficacy of physeal bar resection with guided growth in the treatment of physeal arrest with angular limb deformity. Sci. Rep..

[14] Fu G., Wang W., Dong Y.F., Lv X.M., Yang Z. (2019). Treatment of Post-traumatic Pediatric Ankle Varus Deformity with Physeal Bar Resection and Hemi-Epiphysiodesis. Curr. Med. Sci..

[15] Su A.W., Larson A.N. (2015). Pediatric Ankle Fractures: Concepts and Treatment Principles. Foot Ankle Clin..

[16] Tunçez M., Kazımoğlu C. (2022). Treatment of ankle varus deformity due to physeal bar formation: A case report. Turk. J. Trauma Emerg. Surg..

[17] Berson L., Davidson R.S., Dormans J.P., Drummond D.S., Gregg J.R. (2000). Growth disturbances after distal tibial physeal fractures. Foot Ankle Int..

[18] Lalonde K.A., Letts M. (2005). Traumatic growth arrest of the distal tibia: A clinical and radiographic review. Can. J. Surg..

[19] Yoshida T., Kim W.C., Tsuchida Y., Hirashima T., Oka Y., Kubo T. (2008). Experience of bone bridge resection and bone wax packing for partial growth arrest of distal tibia. J. Orthop. Trauma.

[20] Basiglini L., Aulisa A.G., Bandinelli D., Toniolo R.M., Falciglia F. (2022). Bone wax in the treatment of partial epiphysiodesis of distal femoral growth plate: Case report at 10-year follow-up. Front. Surg..

[21] Xiao H., Li M., Tan Q., Ye W., Wu J., Mei H., Zhu G., Yan A. (2023). Physeal bar resection by modified arthroscopically assisted surgery in a closed osteocavity. Front. Pediatr..

[22] Kim H.T., Lim K.P., Jang J.H., Ahn T.Y. (2015). Resection of a physeal bar with complete transverse osteotomy at the metaphysis and Ilizarov external fixation. Bone Jt. J..

[23] Kang H.G., Yoon S.J., Kim J.R. (2010). Resection of a physeal bar under computer-assisted guidance. J. Bone Jt. Surg. Br. Vol..

[24] Pharr Z.K., Roaten J.D., Moisan A., Kelly D.M., Sawyer J.R. (2018). Use of a Core Reamer for the Resection of a Central Distal Femoral Physeal Bone Bridge: A Novel Technique with 3-Year Follow-up. Am. J. Orthop..

